# Supplementary material for: Getting to the Meat of It: The Effects of a Captive Diet upon the Skull Morphology of the Lion and Tiger
Source: Animals (Basel). 2023 Nov 22;13(23):3616. doi: 10.3390/ani13233616 (PMC10705091; doi:10.3390/ani13233616)
Supplement: Supplementary file 1 [file animals-13-03616-s001.zip › Supplementary Information.pdf]

Supplementary Information

Contents

Extended Acknowledgements

Table S1: Measurements taken corresponding to the subsequent definition of points, definition of measurements, and *Figure S1*. Measurements with a mean coefficient of variation > 1, identified either from this study, or from **(Barnett *et al.*, 2008)** are removed from imputation and analysis (**identified in bold**). Cranial volume is kept due to its known discriminatory power between captive and wild lions **(Saragusty *et al.*, 2014)**, and all canine measurements are removed due to apparent uncertainties identified during the measurement process.

Definition for points (Barnett *et al.*, 2008):

Definition for measurements (Barnett *et al.*, 2008)

Figure S1: Figure from **(Barnett *et al.*, 2008)** which corresponds to the preceding *Table S1* of measurements, **Definition of points**, and **Definition of measurements** sections

Figure S2: The effect of multiple imputation of chained equations (MICE) on a Principal Component Analysis of the imputed data. The figure displays 95% confidence ellipses associated with each imputed specimen. Specimens with no missing data are shown in black, without confidence ellipses.

Table S2: t-test p-values between captive and wild samples of scaled variables, when analysed together (All data), for female and male lions (FL, ML), female and male tigers (FT,MT), female and male Amur tiger (FTA + MTA), female and male Sumatran tiger (FTS, MTS) and female and male northern lion(FLN, MLN). p-values below 0.0009 Bonferroni correction are highlighted in red.

Figures S3-S6: Loading contributions for shape principal components when male and female lions and tigers have been analysed together and separately for male and female lions and tigers. The distributions of captive and wild specimens are shown across sPCs using violin plots.

Figure S7-S12: Variation of size independent (scaled) variables by captivity status for measurements when the data is split by geographical grouping. Measurements larger than zero are larger than average, and measurements smaller than zero are below average for a given sized skull. Measurements which differ significantly by captivity status are highlighted by which population mean is larger. Significance is determined using t-tests (Table S2) based on values of 0.05 and after a Bonferroni Correction, 0.0009.

Figure S13: Measurements of tooth length and isosize against captive lions and tigers of known age. There is no relationship between isosize and age (Spearman's correlation p=0.643). Tooth length decreases significantly with age in Pm4 length (maxilla) p=0.037 and in Pm4 length (mandible) p=0.002. Whilst not significant, M1 length (mandible) also decreases with age (p=0.325). This suggests that tooth wear may have affected tooth measurements, and that smaller tooth size in captive big cats may in part be explained by their longer life expectancy when compared with those from the wild.

Extended Acknowledgements

We thank the European Union SYNTHESYS programme for its financial support to NY for visiting Musée Royale de l'Afrique Centrale, Tervuren, Belgium, Muséum National d'Histoire Naturelle, Paris, France, and Museum für Naturkunde, Berlin, Germany. We also thank Dafne Hills, Paula Jenkins, Louise Tomsett, Richard Sabin at Natural History Museum, London, Malcolm Harman at Powell-Cotton Museum, Kent, Malgosia Nowak-Kemp at Natural History Museum, University of Oxford, Oxford, Claudio Sillero, UK, Robert Asher, Peter Giere, Wolf-Dieter Heinrich, Irene Thomas at Museum für Naturkunde, Berlin, Katrin Krohmann, Thomas Martin at Forschungsinstitut und Naturmuseum Senckenberg, Frankfurt, Alexander Daasch, Frederik Jessen, Thomas Kaiser at Zoologisches Museum Hamburg, Stefan Merker, Doris Mörike at Naturkundemuseum Stuttgart, Stuttgart, Germany, Alexander Bibl, Frank Zachos at Naturhistorisches Museum, Vienna, Austria, Marianne Haffner, Martina Schenkel at Museumsleiterin Zoologisches Museum, Zurich, Switzerland, Gábor Csorba, Mihály Gasparik, Tamás Görföl at Magyar Természettudományi Múzeum, Budapest, Hungary, Bo Fernholm, Daniela Kalthoff, Olavi Grönwall, Lars Werdelin at Naturhistoriska Riksmuseet, Stockholm, Sweden, Tom Gilbert, Kristian Gregersen, Daniel Johansson, Eline Lorenzen at Statens Naturhistoriske Museum, Københavns Universitet, Copenhagen, Denmark, Vincent Nijman, Adri Rol, Peter J. H. van Bree at Zoölogisch Museum, University of Amsterdam, Chris Smeenk, Hein van Grouw at Naturalis Biodiversity Centre, Leiden, Leiden, Emmanuel Gilissen, Wim Wendelen at Musée Royale de l'Afrique Centrale, Tervuren, Belgium, Cécile Callou, Jacques Cuisin, Francis Renoud, Daniel Robineau, Michel Tranier at Muséum National d'Histoire Naturelle, Paris, Marie-Dominique Wandhammer, Virginia Rakotondrahaja at Musée Zoologique, Strasbourg, France, Angel Garvía at Museo Nacional de Ciencias Naturales, Madrid, Spain, Cristiane Bastos-Silveira at Museu Nacional de História Natural e da Ciência, Lisbon, Portugal, Shaw Badenhorst, Duncan MacFadyen, Wynand van Zyl at Ditsong National Museum of Natural History, Pretoria, Nigel Bennett at Mammal Research Institute at University of Pretoria, Pretoria, Sifelani Jirah, Viviene Williams, Bernhard Zipfel at University of the Witwatersrand, Johannesburg, Gregory Brett at East London Museum, East London, Gillian Watson at Port Elizabeth Museum Complex, Port Elizabeth, Denise Hamerton at Iziko South African Museum, Cape Town, Fred Kigozi at Amathole Museum, King William's Town, South Africa, Woody Cotteril, Tsitsi Maponga, Kith Mkhwananzi, Melusi Sibanda at Natural History Museum, Bulawayo, Zimbabwe, Vitaliy Kascheev, Amankul Bekenov at Institute of Zoology, Almaty, Vitaliy Gromov,

Institute of Soil Science, Almaty, Kazakhstan. Peng He, Fuwen Wei at Institute of Zoology, Chinese Academy of Sciences, Beijing, China, Sergei Kruskop, Vladimir Lebedev, Igor Ya. Pavlinov at Zoological Museum of Moscow State University, Moscow, Alexei Abramov, Zoological Institute, Russian Academy of Sciences, St. Petersburg, Elena I. Zholnerovskaya, Natasha V. Lopatina at Siberian Branch of Russian Academy of Sciences, Novosibirsk, Irina Volvenko at Zoological Museum, Far Eastern Federal University, Vladivostok, Mikhail Tiunov, Olga Uphyrkina at Federal Scientific Centre for the Biodiversity of Terrestrial Biota of East Asia, Far Eastern Branch, Russian Academy of Sciences, Vladivostok, Alexander Myslenkov, at Lazovsky Nature Reserve, Lazo, Russia, for their kind support for the access to their collections.

Table S1: Measurements taken corresponding to the subsequent definition of points, definition of measurements, and *Figure S1*. Measurements with a mean coefficient of variation > 1, identified either from this study, or from **(Barnett *et al.*, 2008)** are removed from imputation and analysis (**identified in bold**). Cranial volume is kept due to its known discriminatory power between captive and wild lions **(Saragusty *et al.*, 2014)**, and all canine measurements are removed due to apparent uncertainties identified during the measurement process.

**Continuous Measurements**	**Mean Coefficient of variation**	**Coefficient of variation** (Barnett et al., 2008)
Cranial volume (ml)	1.00 (± 0.305)	0.35 (± 0.084)
Frontal breadth [21]	0.05 (± 0.016)	0.08 (± 0.021)
Greatest length [A-B]	0.31 (± 0.193)	0.04 (± 0.012)
Condylobasal length [A-C]	0.13 (± 0.051)	0.03 (± 0.002)
Palate-inion [V-B]	0.20 (± 0.014)	0.14 (± 0.039)
Nasal-inion [E-B]	0.04 (± 0.015)	0.14 (± 0.035)
Facial length [A-G]	0.12 (± 0.045)	0.17 (± 0.034)
Head length [G-B]	0.07 (± 0.017)	0.08 (± 0.015)
Bizygomatic breadth [24]	0.03 (± 0.01)	0.04 (± 0.009)
Zygomatic length [K-M]	0.29 (± 0.116)	0.11 (± 0.028)
Zygomatic length anterior [K-L]	0.37 (± 0.066)	0.3 (± 0.048)
Zygomatic length posterior [L-M]	0.44 (± 0.08)	0.31 (± 0.064)
Orbit vertical [4]	0.24 (± 0.074)	0.5 (± 0.195)
Orbit horizontal [3]	0.55 (± 0.142)	0.94 (± 0.198)
Postorbital bar [2]	0.34 (± 0.128)	0.28 (± 0.043)
Facial length anterior [A-E]	0.6 (± 0.152)	0.59 (± 0.085)
Facial length posterior [E-G]	0.12 (± 0.019)	0.73 (± 0.166)
Sagittal crest [H-B]	0.25 (± 0.067)	0.23 (± 0.064)
Cranial height-I [N-H]	0.5 (± 0.124)	0.19 (± 0.027)
Cranial height-II [N-H']	0.46 (± 0.116)	0.4 (± 0.116)
Cranial height-III [N-H"]	0.3 (± 0.045)	0.33 (± 0.078)
Cranial height -IV [N-B]	0.18 (± 0.067)	0.34 (± 0.049)
Interorbital breadth [20]	0.27 (± 0.203)	0.1 (± 0.016)
Postorbital breadth [22]	0.17 (± 0.062)	0.15 (± 0.073)
Nasal length-I [D-F]	0.34 (± 0.091)	0.33 (± 0.144)
**Nasal length-II [S-F]**	**1.23 (± 0.251)**	0.42 (± 0.202)
Nasal breadth [D-D]	0.64 (± 0.144)	0.57 (± 0.11)
Breadth between infra orbital foramina [19]	0.25 (± 0.071)	0.17 (± 0.055)
Rostral depth-I [1]	0.62 (± 0.177)	0.75 (± 0.14)
Rostral depth-II [E - most posterior end of canine alveolus]	0.45 (± 0.05)	0.53 (± 0.057)
Rostral breadth [17]	0.13 (± 0.025)	0.16 (± 0.036)
Nasal aperture [18]	0.53 (± 0.071)	0.8 (± 0.448)
Upper jaw [A-U]	0.12 (± 0.029)	0.1 (± 0.018)
Palate length [T-V]	0.27 (± 0.065)	0.14 (± 0.041)
Palate breadth-I [29]	0.22 (± 0.04)	0.14 (± 0.034)
Palate breadth-II [28]	0.47 (± 0.103)	0.16 (± 0.045)
Canine - Pm^4 (alveolus - alveolus)	0.42 (± 0.116)	0.46 (± 0.136)
Pm^2 - Pm^4 (alveolus - alveolus)	0.43 (± 0.153)	0.25 (± 0.129)

**Upper canine height [5]**	**2.76 (± 0.717)**	0.36 (± 0.18)
**Upper canine diameter antero-posterior [25]**	0.69 (± 0.274)	0.53 (± 0.16)
**Upper canine diameter medio-lateral [26]**	0.88 (± 0.306)	0.57 (± 0.171)
**Upper canine alveolus diameter antero-posterior**	0.99 (± 0.49)	1 (± 0.233)
**Upper canine alveolus diameter medio-lateral**	**1.77 (± 0.36)**	2.02 (± 0.702)
Pm^4 length [6]	0.68 (± 0.378)	0.18 (± 0.04)
**Pm^4 breadth-I [27]**	**1.77 (± 1.01)**	0.83 (± 0.153)
**Pm^4 breadth-II [27]**	**1.78 (± 0.836)**	0.37 (± 0.092)
Mastoid breadth [23]	0.03 (± 0.01)	0.04 (± 0.009)
Skull height-I [W-B]	0.23 (± 0.038)	0.79 (± 0.41)
Skull height-II [7]	0.1 (± 0.028)	0.55 (± 0.32)
**Foramen magnum breadth**	**1.78 (± 1.139)**	0.16 (± 0.022)
Foramen magnum height [greatest distance: usually oblique]	0.5 (± 0.155)	0.65 (± 0.188)
Occipital condyles breadth [33]	0.04 (± 0.008)	0.08 (± 0.043)
**Tympanic bulla length [31]**	**1.58 (± 0.923)**	0.79 (± 0.137)
**Tympanic bulla breadth-I [30]**	0.89 (± 0.401)	**1.42 (± 0.559)**
**Tympanic bulla breadth-II [32]**	**2.22 (± 0.661)**	**2.96 (± 0.269)**
Mandible length [O-Q]	0.08 (± 0.031)	0.18 (± 0.042)
Mandible length coronoid process [O-Q']	0.05 (± 0.023)	0.19 (± 0.058)
Mandible length angular process [O-Q"]	0.1 (± 0.052)	0.07 (± 0.019)
Mandible height [13]	0.34 (± 0.152)	0.23 (± 0.069)
Mandible height angular process [14]	0.91 (± 0.344)	0.57 (± 0.182)
Mandible height coronoid process [15]	0.35 (± 0.126)	0.16 (± 0.04)
Maximum width of the mandibular condyle [16]	0.09 (± 0.023)	0.08 (± 0.02)
Mandible depth-I [11]	0.6 (± 0.176)	0.51 (± 0.237)
Mandible depth-II [12]	0.41 (± 0.262)	0.2 (± 0.043)
Canine - M_1 (alveolus - alveolus)	0.26 (± 0.036)	0.27 (± 0.088)
Pm_3 - M_1 (alveolus - alveolus)	0.52 (± 0.079)	0.3 (± 0.077)
**Lower canine height [8]**	0.45 (± 0.075)	**1.33 (± 0.463)**
**Lower canine diameter antero-posterior**	0.68 (± 0.269)	0.78 (± 0.222)
**Lower canine diameter medio-lateral**	**1.33 (± 0.222)**	1.56 (± 0.29)
**Lower canine alveolus diameter antero-posterior**	**1.11 (± 0.393)**	**1.03 (± 0.189)**
**Lower canine alveolus diameter medio-lateral**	**2.17 (± 0.269)**	**1.72 (± 0.674)**
Pm_4 length	0.71 (± 0.108)	0.13 (± 0.022)
**Pm_4 breadth (largest breadth usually towards the posterior end**	**2.06 (± 0.302)**	0.54 (± 0.178)
M_1 length [9]	0.48 (± 0.265)	0.95 (± 0.342)
**M_1 breadth (largest breadth usually around the middle)**	**1.97 (± 0.744)**	0.4 (± 0.117)

Definition for points (Barnett *et al.*, 2008):

A. Prosthion: the most anterior point of the skull
B. Inion: the most posterior point of the skull
C. The line connecting the most posterior points of the occipital condyles
D. The most anterior points of the nasals
E. The highest points on the vaults of the anterior ends of the dorsal parts of the nasals
F. The most posterior point of the inter nasal suture
G. The point where the line connecting the most outer points of the postorbital process of the frontal meets the mesion
H. Bregma: where the coronal suture meets sagittal suture (if the sagittal crest is very well developed, use the place where the coronal suture reaches the top of the sagittal crest in the mesion)
H'. One third distance point between bregma and inion
H''. Two third distance point between bregma and inion
I. Vertical lines including the most outer points of the alveoli of I^3s
J. The most anterior point of alveolus of the upper canine
K. The most dorsal point of infraorbital foramen (in case if there are more than one foramen, the most dorsal point of the foramina)
L. The most outer point of the zygomatic arch (usually just above the malar - temporal suture)
M. A point where a vertical section including the most dorsal point of the auditory meatus cuts the outer curve of the zygomatic process of the temporal
N. The most dorsal point of the auditory meatus
O. Pogonion: the most anterior point of mandible on the inter mandible suture
P. The most ventral point around the angular process. If it is not obvious, use the point where the extended line of the middle line of inferior notch crosses the ventral edge of angular process as being shown in *Figure S1*. This may sound difficult, but in practice there is little problem and a subtle difference of the position of "P" does not seem to affect the result.
Q. The point where the extended line of the ventral end of superior notch crosses the posterior edge of condyle (approximately the middle of condyle)
Q'. The furthest point on the coronoid process from the pogonion
Q". The furthest point on the angular process from the pogonion
R. The line connecting the highest points on the vaults of the anterior ends of dorsal parts of nasals
S. The most anterior point of the inter nasal suture. Often the inter nasal suture of some skulls may be slightly opened towards the anterior end, and it may be difficult to assess where "S" is. In such case, ignore the part where the inner lines of the nasals forms a sallow angle (almost parallel) to the mesion, and find the point where the angle starts to change.
T. Orale: the point where the line connecting the most posterior points of I^1 alveoli meets the mesion
U. The middle point along the posterior part of the alveolus of M^1
V. The most posterior point of the palate on the mesion
W. The most ventral point in the mesion between occipital condyles

Definition for measurements (Barnett *et al.*, 2008)

1. The distance from R to the most posterior point of the upper canine alveolus.
2. The shortest distance between the most dorsal point of the malar - temporal suture and the most ventral edge of the zygomatic arch.
3. The distance between the tip of the postorbital process of malar and the point where lacrimal - frontal suture crosses the edge of orbit.
4. The longest distance between the tip of postorbital process of the frontal and the edge of the orbit (if the tip of postorbital process of the frontal is not pointy enough, use the middle point of the rounded tip).
5. The distance along the buccal surface of an upper canine between the unworn tip and the point on the cemento - enamel junction where the medio-lateral breadth of the canine becomes its maximum.
6. The distance between the most anterior point of the buccal part of Pm^4 and the most posterior point of the tooth.
7. The greatest distance between W and the sagittal crest around its posterior end. In most case, it is easy to measure this distance. However, if the sagittal crest of the skull is very well developed, measure the distance between W and the sagittal crest around the middle point between H” and B.
8. The distance along the buccal surface of a lower canine between the unworn tip and the point on the cemento - enamel junction where the medio-lateral breadth of the canine becomes its maximum.
9. The distance between most anterior and posterior points of M1.
10. The distance between the most anterior point of canine alveolus and the most posterior point of M^1 alveolus.
11. The smallest of the greatest diameter of the section which cuts the mandible in front of the most anterior point of Pm^3 alveolus: usually an oblique measurement like the one shown in *Figure S1*.
12. The smallest of the greatest diameter of the section which cuts the mandible just behind the most posterior point of M^1 alveolus: usually a vertical or slightly oblique measurement shown in the *Figure S1*.
13. The greatest distance between P and the dorsal part of coronoid process.
14. The greatest distance between P and the dorsal part of the condyle just outside the place where the superior notch crosses the condyle: usually an oblique measurement shown in *Figure S1*.
15. The greatest distance between the ventral part of condyle just inside the place where the condyle meets the inferior notch and the dorsal part of the coronoid process: usually an oblique measurement shown in *Figure S1* – in most cases use the point on the coronoid process that was used for measurement 13.
16. Maximum width of the mandibular condyle.
17. The greatest breadth of the rostrum just above the canine alveoli.
18. The breadth of the nasal aperture above the most outer points of the I^3 alveoli.
19. The smallest distance between the infraorbital foramina.
20. Interorbital breadth: the smallest distance between the orbits.
21. The distance between the most outer points of the postorbital process of the frontal.
22. Postorbital breadth: the smallest breadth of the postorbital constriction.
23. Mastoid breadth: the distance between the most outer points of the mastoidal processes.
24. The distance between zygions: the most outer points of the mastoidal processes.

25. The greatest antero-posterior length of an upper canine at the cemento-enamel junction: the smaller diameter of the canine.
26. The greatest medio-lateral length of an upper canine at the cemento-enamel junction: the smaller diameter of the canine.
27. Pm^4-I: between the inner process and the most anterior outer process, and Pm^4-II: between the former and the second most anterior outer process of the tooth.
28. The smallest distance between the M^1 alveoli.
29. The greatest distance between the Pm^4 alveoli.
30. The distance between the most anterior/inner meeting point between the tympanic bulla and the external auditory meatus and the most anterior meeting point between the tympanic bulla and the foramen lacerum posterius.
31. The greatest length of the tympanic bulla excluding styloid process and other processes attached to the tympanic bulla: fix one end of the calliper at the point where the foramen lacerum medius meets the most prominent styloid process, and measure the greatest distance between that point and the posterior part of the tympanic bulla.
32. The greatest mediolateral distance of the vault of the tympanic bulla: the smaller diameter of the vault of the tympanic bulla.
33. The greatest breadth of the occipital condyles.

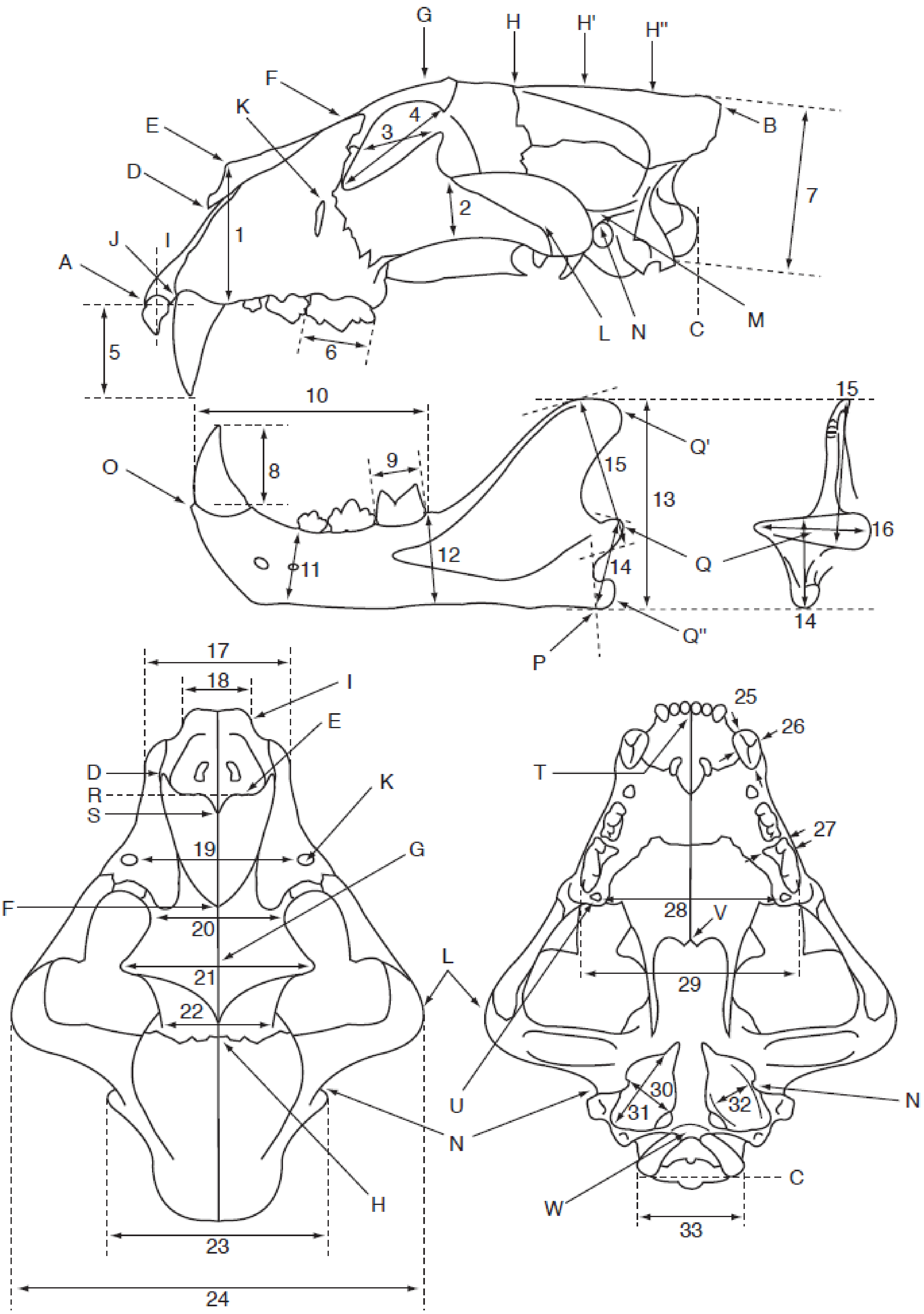

Figure S1: Figure from **(Barnett *et al.*, 2008)** which corresponds to the preceding *Table S1* of measurements, **Definition of points**, and **Definition of measurements** sections.

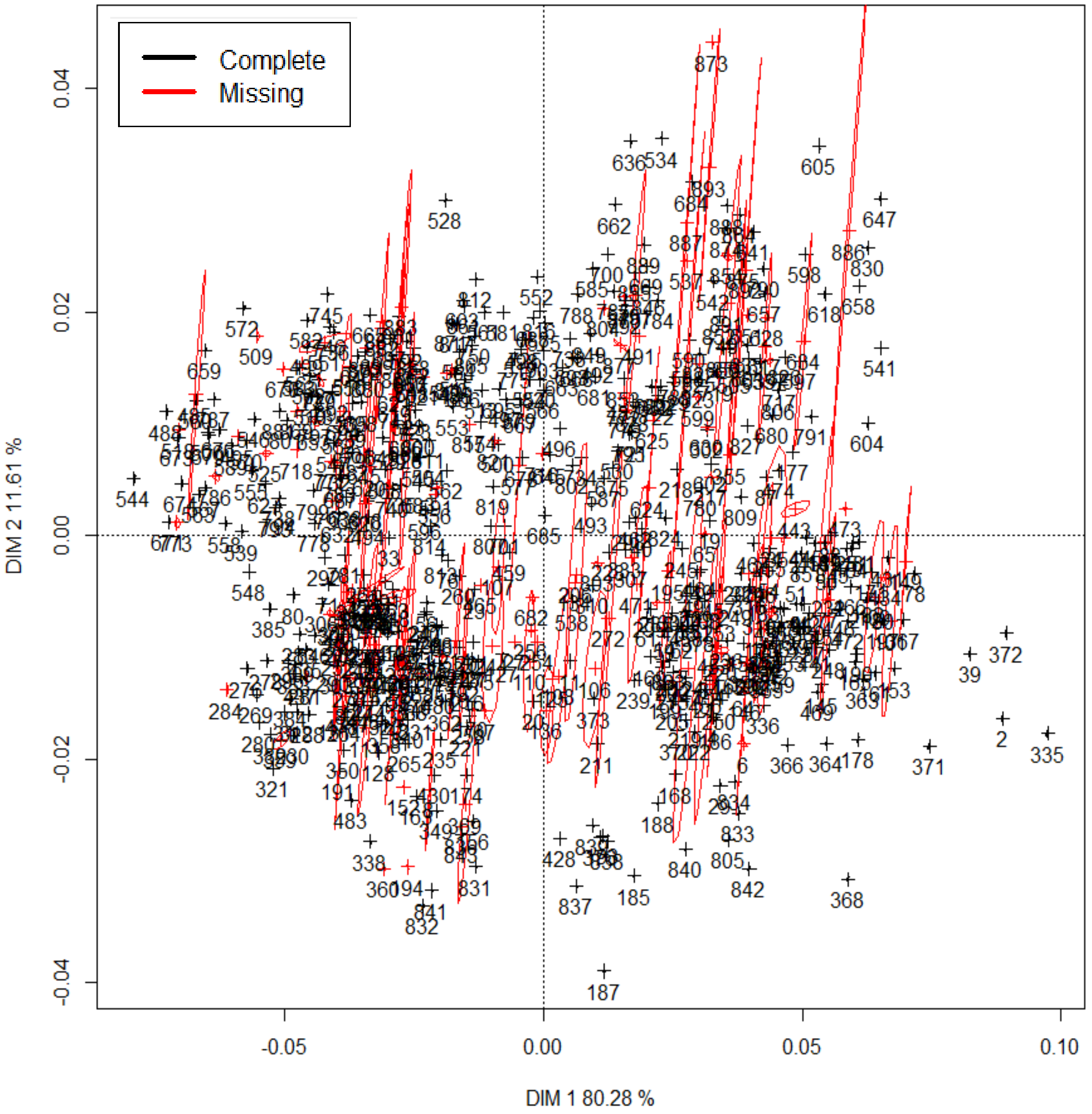

Figure S2: The effect of multiple imputation of chained equations (MICE) on a Principal Component Analysis of the imputed data. The figure displays 95% confidence ellipses associated with each imputed specimen. Specimens with no missing data are shown in black, without confidence ellipses.

Table S2: t-test p-values between captive and wild samples of scaled variables, when analysed together (All data), for female and male lions (FL, ML), female and male tigers (FT,MT), female and male Amur tiger (FTA + MTA), female and male Sumatran tiger (FTS, MTS) and female and male northern lion(FLN, MLN). p-values below 0.0009 Bonferroni correction are highlighted in red.

	All Data	FL	ML	FT	MT	FTA	MTA	FTS	MTS	FLN	MLN
Cranial volume (ml)	0.07	0.00	0.00	0.00	0.01	0.00	0.09	0.66	0.03	0.08	0.03
Frontal breadth	0.00	0.16	0.01	0.00	0.00	0.00	0.00	0.03	0.78	0.04	0.40
Greatest length	0.76	0.20	0.70	0.74	0.12	0.75	0.17	0.42	0.86	0.68	0.50
Condylobasal length	0.01	0.01	0.04	0.96	0.51	0.54	0.67	0.74	0.03	0.10	0.08
Palate-inion	0.38	0.98	0.11	0.67	0.81	0.99	0.64	0.68	0.38	0.42	0.14
Nasal-inion	0.76	0.10	0.01	0.28	0.00	0.35	0.92	0.09	0.50	0.12	0.00
Facial length	0.09	0.23	0.47	0.96	0.77	0.01	0.03	0.47	0.16	0.93	0.09
Head length	0.90	0.16	0.00	0.00	0.00	0.04	0.00	0.01	0.20	0.02	0.02
Bizygomatic breadth	0.00	0.00	0.00	0.00	0.05	0.01	0.35	0.03	0.34	0.00	0.00
Zygomatic length	0.01	0.05	0.57	0.74	0.33	0.81	0.83	0.30	0.00	0.46	0.69
Zygomatic length anterior	0.00	0.01	0.00	0.20	0.37	0.20	0.23	0.01	0.09	0.14	0.08
Zygomatic length posterior	0.00	0.00	0.00	0.00	0.89	0.27	0.51	0.40	0.65	0.00	0.00
Orbit vertical	0.06	0.98	0.00	0.91	0.81	0.05	0.34	0.29	0.09	0.70	0.00
Orbit horizontal	0.00	0.09	0.40	0.23	0.43	0.17	0.59	0.53	0.60	0.11	0.49
Postorbital bar	0.42	0.19	0.87	0.79	0.42	0.22	0.92	0.10	0.19	0.08	0.18
Facial length anterior	0.37	0.86	0.04	0.00	0.01	0.00	0.01	0.00	0.03	0.75	0.17
Facial length posterior	0.00	0.02	0.04	0.02	0.00	0.39	0.81	0.33	0.90	0.66	0.03
Sagittal crest	0.00	0.02	0.04	0.50	0.71	0.18	0.00	0.12	0.73	0.34	0.27
Cranial height-I	0.73	0.00	0.15	0.39	0.03	0.19	0.00	0.26	0.53	0.19	0.89
Cranial height-II	0.16	0.03	0.05	0.08	0.76	0.63	0.01	0.50	0.18	0.05	0.94
Cranial height-III	0.01	0.29	0.14	0.02	0.70	0.62	0.00	0.52	0.88	0.69	0.45
Cranial height -IV	0.01	0.08	0.07	0.15	0.00	0.00	0.00	0.24	0.13	0.19	0.04
Interorbital breadth	0.00	0.00	0.03	0.33	0.05	0.00	0.00	0.19	0.25	0.00	0.10
Postorbital breadth	0.00	0.01	0.00	0.16	0.19	0.28	0.01	0.02	0.01	0.86	0.02
Nasal length-I	0.02	0.22	0.00	0.00	0.00	0.37	0.02	0.22	0.66	0.56	0.08
Nasal breadth	0.09	0.06	0.06	0.00	0.05	0.12	0.62	0.26	0.82	0.07	0.29
Breadth between infra orbital foramina	0.00	0.00	0.00	0.61	0.00	0.01	0.00	0.03	0.35	0.03	0.19
Rostral depth-I	0.00	0.22	0.67	0.00	0.01	0.00	0.02	0.01	0.00	0.35	0.53
Rostral depth-II	0.00	0.10	0.14	0.00	0.00	0.00	0.00	0.04	0.00	0.36	0.21
Rostral breadth	0.00	0.01	0.00	0.00	0.00	0.00	0.01	0.94	0.00	0.04	0.03
Nasal aperture	0.00	0.11	0.00	0.91	0.01	0.00	0.00	0.57	0.08	0.28	0.01
Upper jaw	0.46	0.41	0.03	0.82	0.00	0.46	0.02	0.24	0.41	0.83	0.05
Palate length	0.00	0.02	0.00	0.71	0.53	0.51	0.42	0.84	0.27	0.14	0.00
Palate breadth-I	0.00	0.00	0.00	0.42	0.10	0.01	0.21	0.62	0.98	0.03	0.00
Palate breadth-II	0.00	0.00	0.00	0.00	0.00	0.55	0.61	0.38	0.13	0.00	0.00
Canine - Pm^4 (alveolus - alveolus)	0.20	0.70	0.60	0.90	0.00	0.23	0.20	0.08	0.83	0.27	0.17
Pm^2 - Pm^4 (alveolus - alveolus)	0.00	0.06	0.00	0.64	0.00	0.16	0.05	0.02	0.06	0.68	0.82
Pm^4 length	0.00	0.00	0.00	0.00	0.02	0.00	0.77	0.03	0.08	0.28	0.31
Mastoid breadth	0.00	0.00	0.00	0.26	0.00	0.07	0.05	0.10	0.10	0.00	0.00
Skull height-I	0.07	0.07	0.02	0.78	0.06	0.09	0.20	0.63	0.20	0.02	0.15
Skull height-II	0.18	0.18	0.45	0.05	0.97	0.31	0.01	0.50	0.23	0.04	0.76
Foramen magnum height	0.00	0.00	0.00	0.23	0.83	0.05	0.72	0.57	0.66	0.07	0.00
Occipital condyles breadth	0.00	0.01	0.00	0.01	0.16	0.00	0.03	0.00	0.03	0.07	0.00
Mandible length	0.20	0.03	0.01	0.89	0.05	0.97	0.68	0.74	0.03	0.05	0.12
Mandible length coronoid process	0.60	0.03	0.48	0.07	0.33	0.50	0.95	0.07	0.56	0.38	0.06
Mandible length angular process	0.00	0.00	0.00	0.23	0.00	0.47	0.49	0.59	0.33	0.14	0.01
Mandible height	0.00	0.00	0.00	0.00	0.00	0.00	0.00	0.57	0.04	0.04	0.01
Mandible height angular process	0.00	0.00	0.00	0.00	0.00	0.18	0.00	0.63	0.11	0.10	0.18
Mandible height coronoid process	0.00	0.18	0.00	0.00	0.00	0.00	0.00	0.53	0.42	0.05	0.01
Width of the mandibular condyle	0.00	0.00	0.00	0.40	0.08	0.65	0.43	0.03	0.36	0.07	0.00
Mandible depth-I	0.00	0.02	0.00	0.00	0.00	0.00	0.01	0.02	0.08	0.19	0.03
Mandible depth-II	0.03	0.21	0.66	0.49	0.24	0.28	0.04	0.11	0.71	0.06	0.79
Canine - M$_1$ (alveolus - alveolus)	0.14	0.36	0.95	0.60	0.00	0.78	0.05	0.01	0.83	0.67	0.66
Pm$_3$ - M$_1$ (alveolus - alveolus)	0.00	0.00	0.01	0.01	0.25	0.20	0.06	0.04	0.11	0.18	0.32
Pm$_4$ length	0.00	0.01	0.00	0.50	0.30	0.01	0.31	0.41	0.38	0.06	0.57
M$_1$ length	0.00	0.00	0.00	0.01	0.26	0.08	0.01	0.05	0.04	0.04	0.00

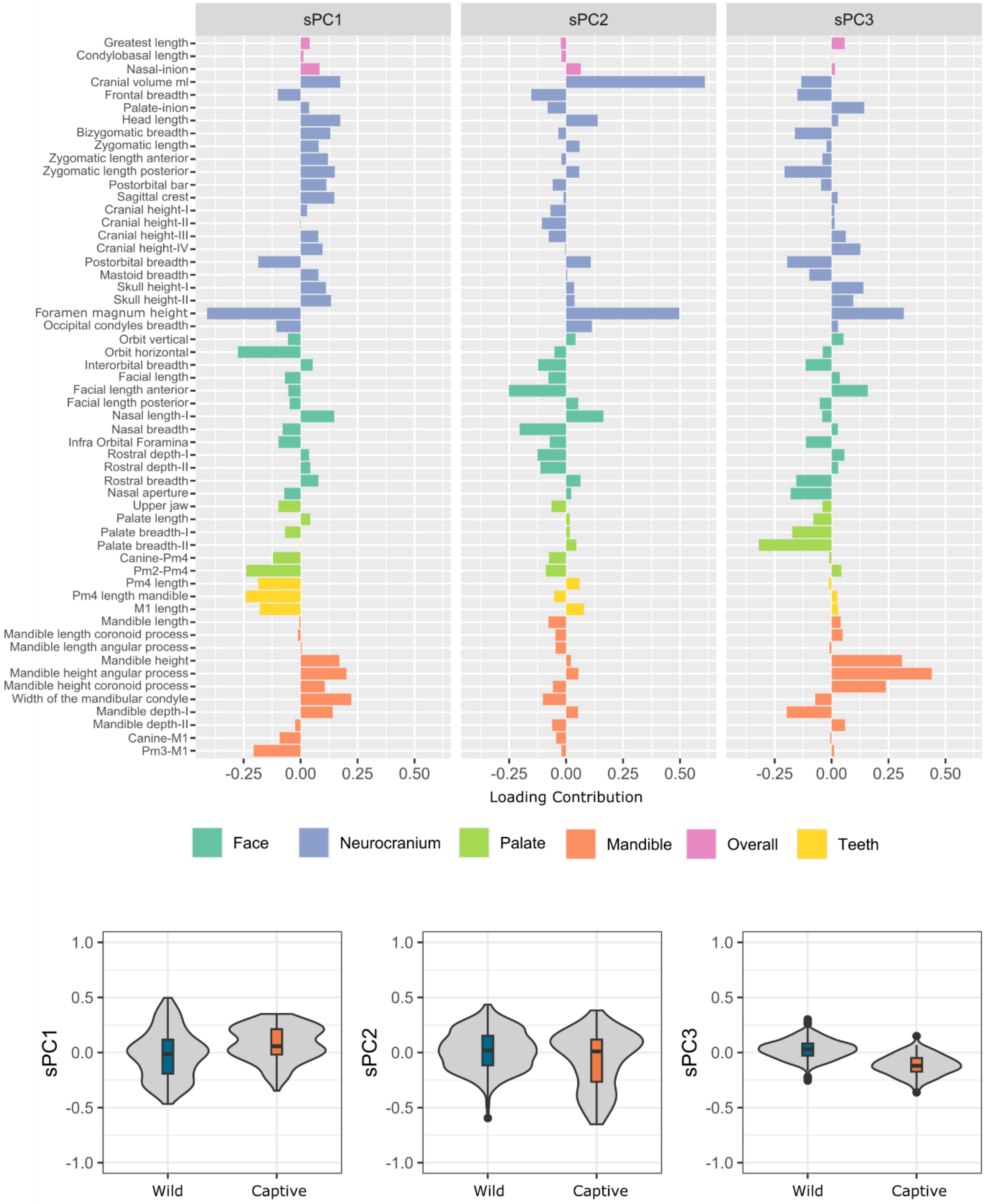

Figure S3: Loading contributions for shape principal components when male and female lions and tigers have been analysed together. The distributions of captive and wild specimens are shown across sPCs using violin plots.

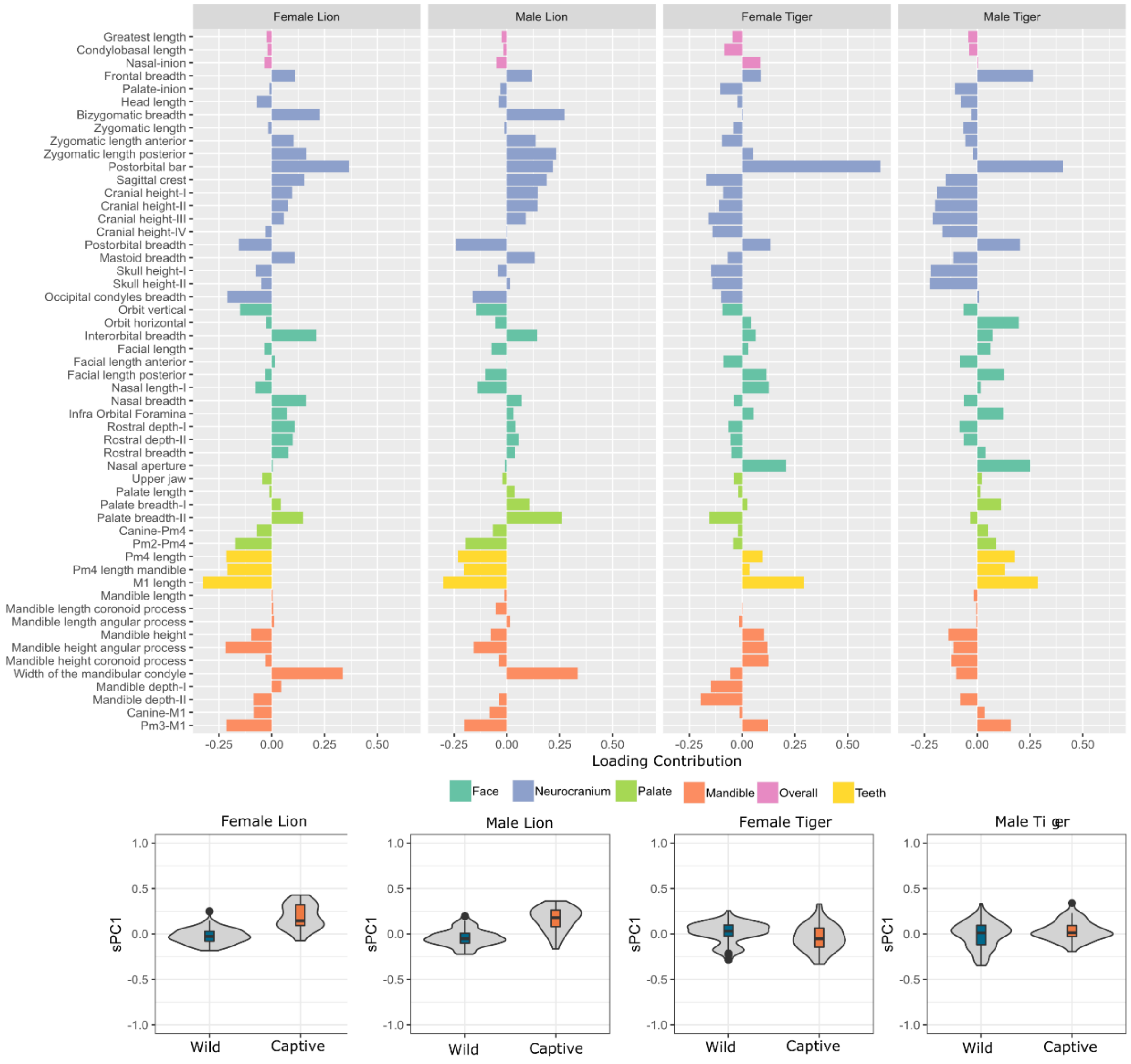

Figure S4: Loading contributions of sPC1 for male and female lions and tigers. The distributions of captive and wild specimens are shown across sPC1 using violin plots.

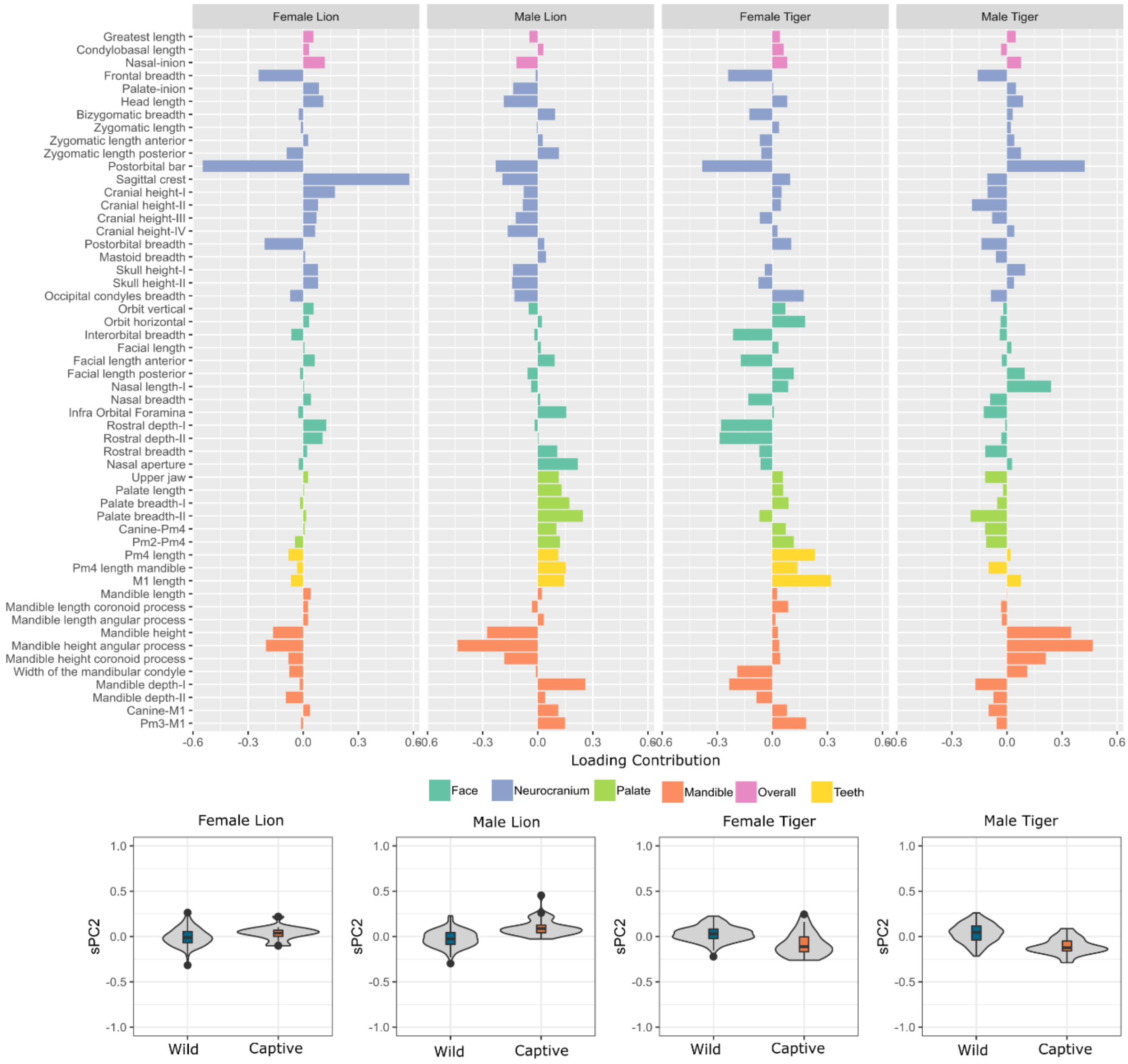

Figure S5: Loading contributions of sPC2 for male and female lions and tigers. The distributions of captive and wild specimens are shown across sPC2 using violin plots.

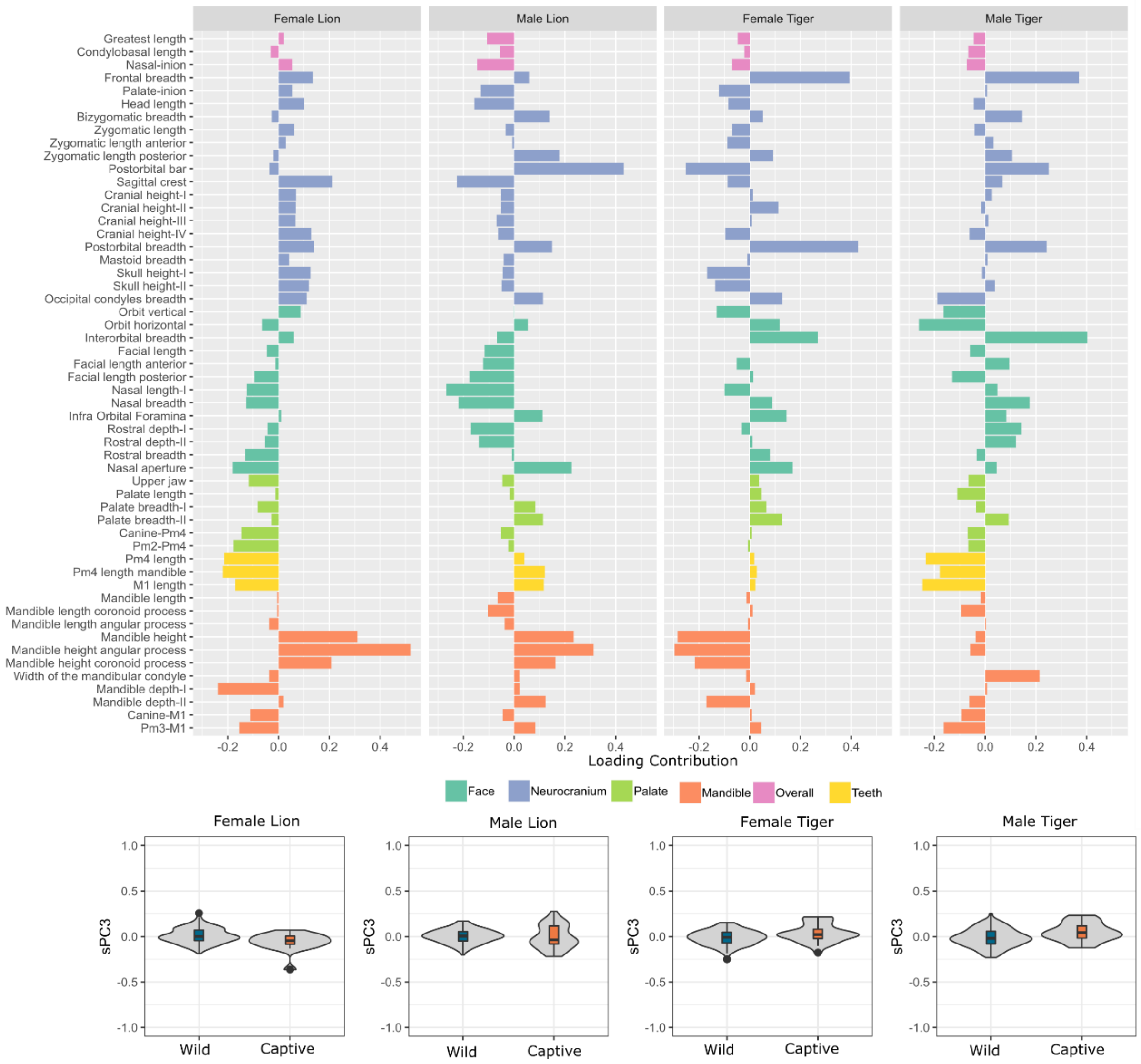

Figure S6: Loading contributions of sPC3 for male and female lions and tigers. The distributions of captive and wild specimens are shown across sPC3 using violin plots.

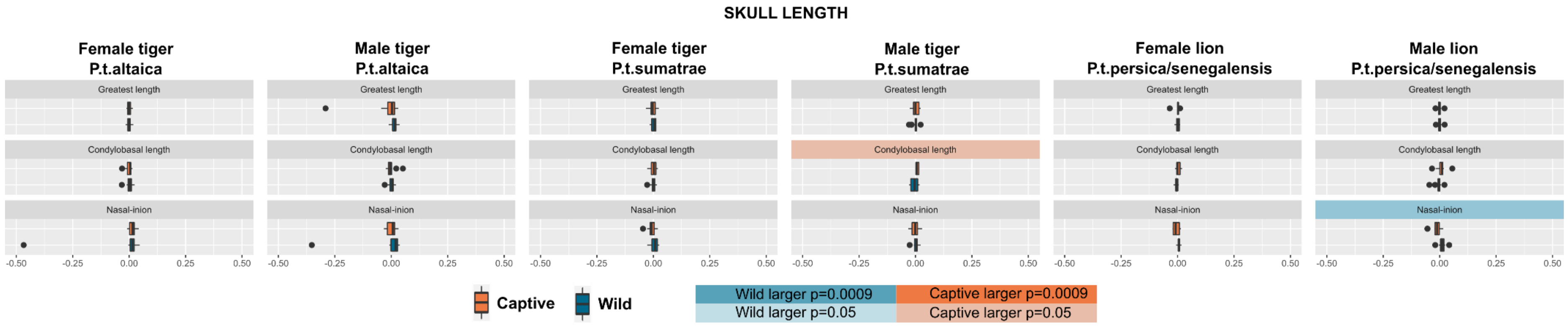

Figure S7: Variation of size independent (scaled) variables by captivity status for measurements of overall skull length when the data is split by nominal grouping. Measurements larger than zero are larger than average, and measurements smaller than zero are below average for a given sized skull. Measurements which differ significantly by captivity status are highlighted by which population mean is larger. Significance is determined using t-tests (Table S2) based on values of 0.05 and after a Bonferroni Correction, 0.0009.

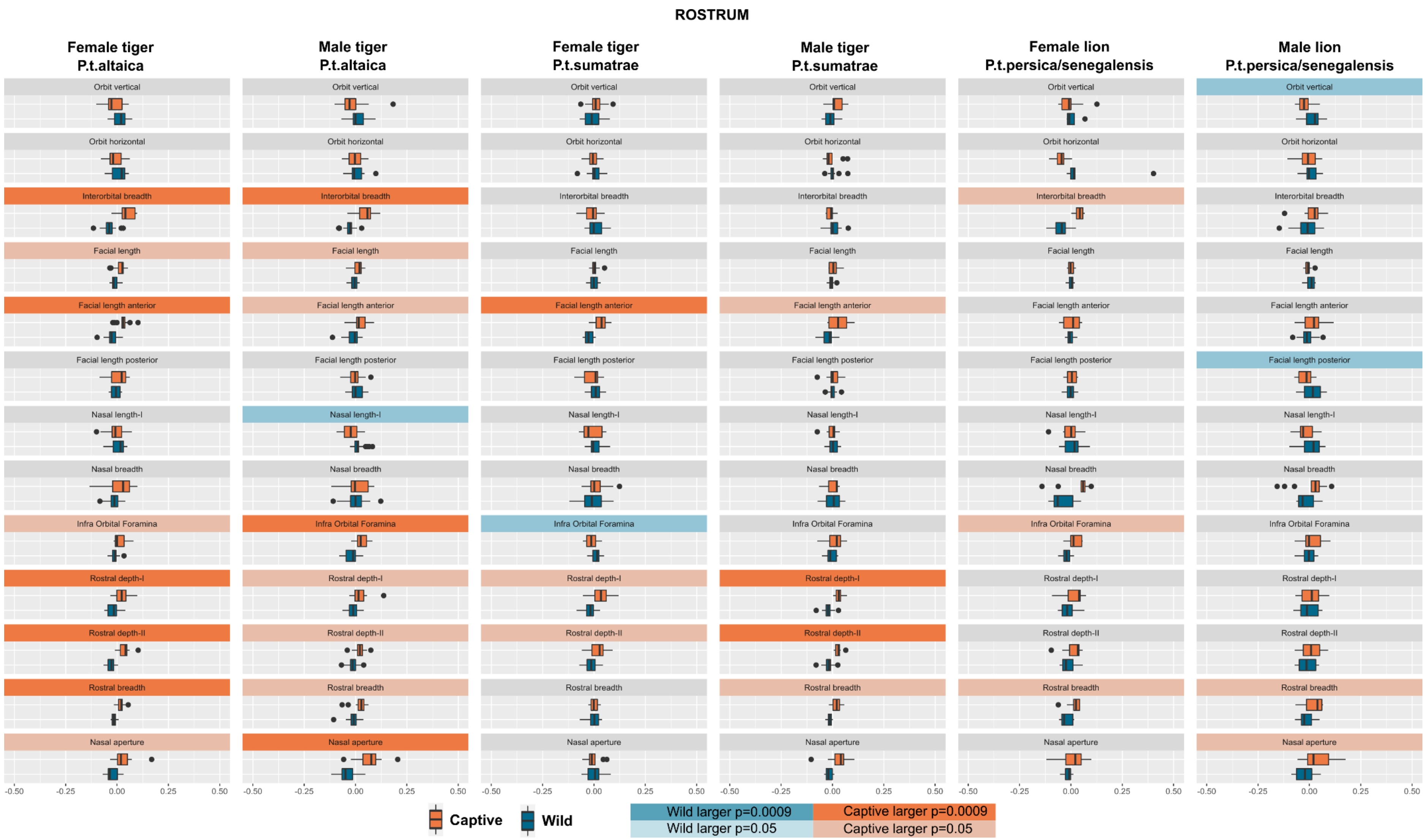

Figure S8: Variation of size independent (scaled) variables by captivity status for measurements of the rostrum when the data is split by nominal grouping. Measurements larger than zero are larger than average, and measurements smaller than zero are below average for a given sized skull. Measurements which differ significantly by captivity status are highlighted by which population mean is larger. Significance is determined using t-tests (Table S2) based on values of 0.05 and after a Bonferroni Correction, 0.0009.

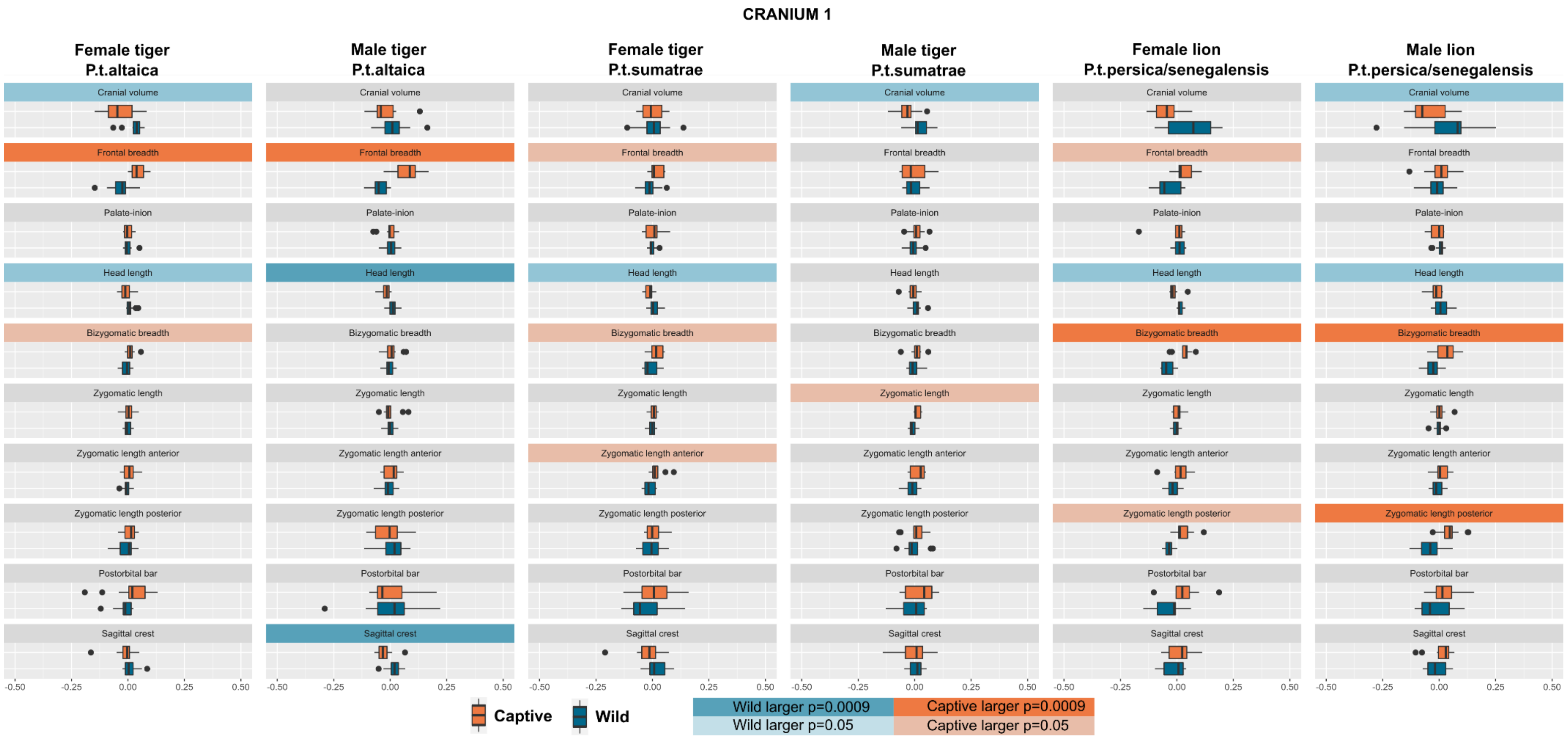

Figure S9: Variation of size independent (scaled) variables by captivity status for measurements of the cranium when the data is split by nominal grouping. Measurements larger than zero are larger than average, and measurements smaller than zero are below average for a given sized skull. Measurements which differ significantly by captivity status are highlighted by which population mean is larger. Significance is determined using t-tests (Table S2) based on values of 0.05 and after a Bonferroni Correction, 0.0009.

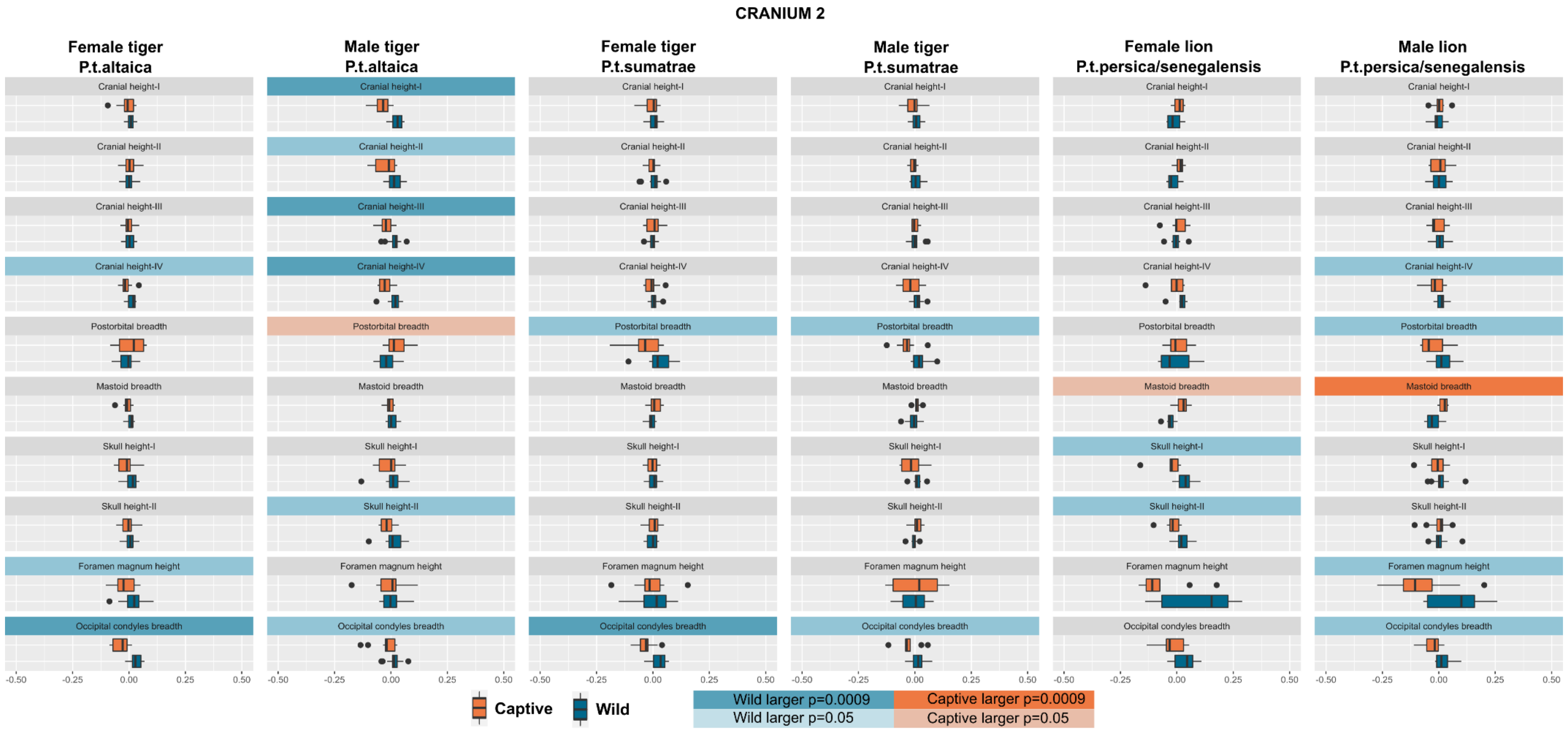

Figure S10: Variation of size independent (scaled) variables by captivity status for measurements of the cranium when the data is split by nominal grouping. Measurements larger than zero are larger than average, and measurements smaller than zero are below average for a given sized skull. Measurements which differ significantly by captivity status are highlighted by which population mean is larger. Significance is determined using t-tests (Table S2) based on values of 0.05 and after a Bonferroni Correction, 0.0009.

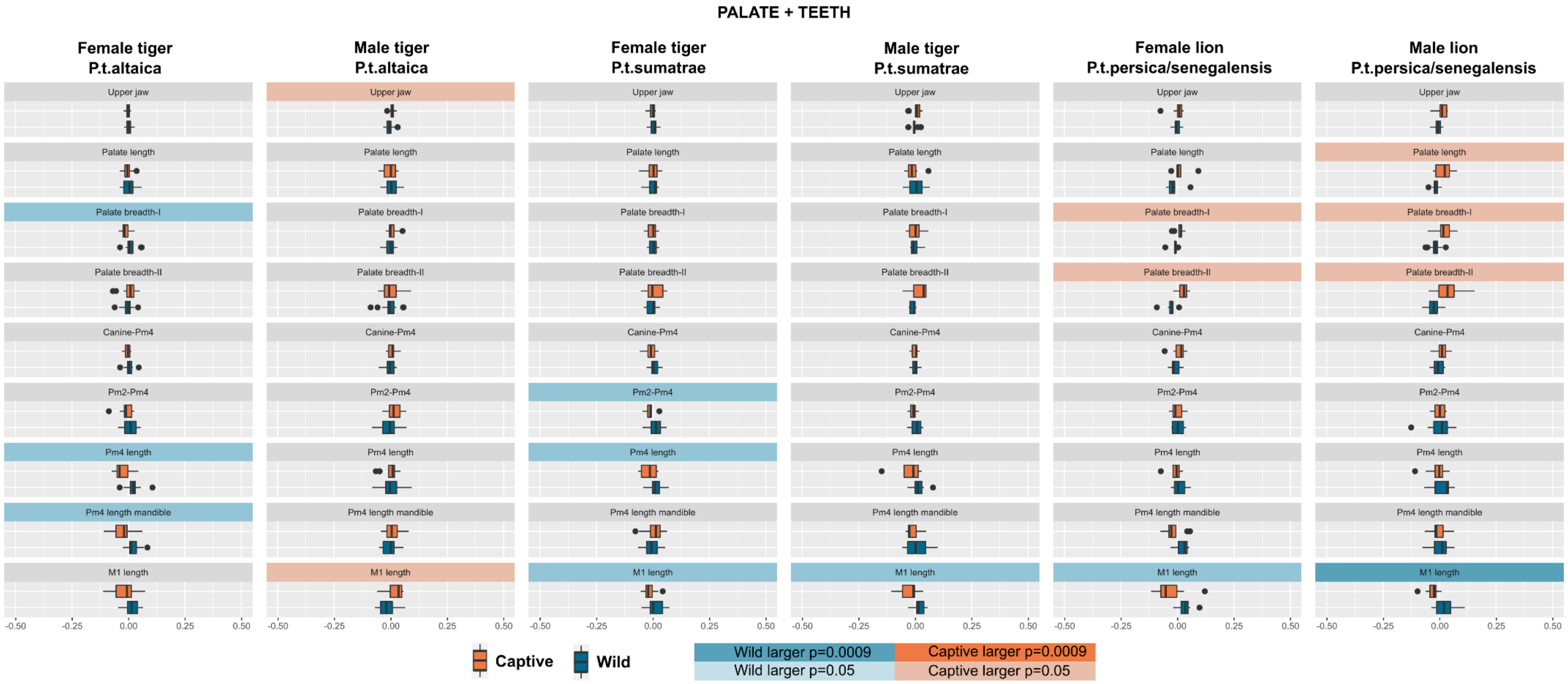

Figure S11: Variation of size independent (scaled) variables by captivity status for measurements of the palate and teeth when the data is split by nominal grouping. Measurements larger than zero are larger than average, and measurements smaller than zero are below average for a given sized skull. Measurements which differ significantly by captivity status are highlighted by which population mean is larger. Significance is determined using t-tests (Table S2) based on values of 0.05 and after a Bonferroni Correction, 0.0009.

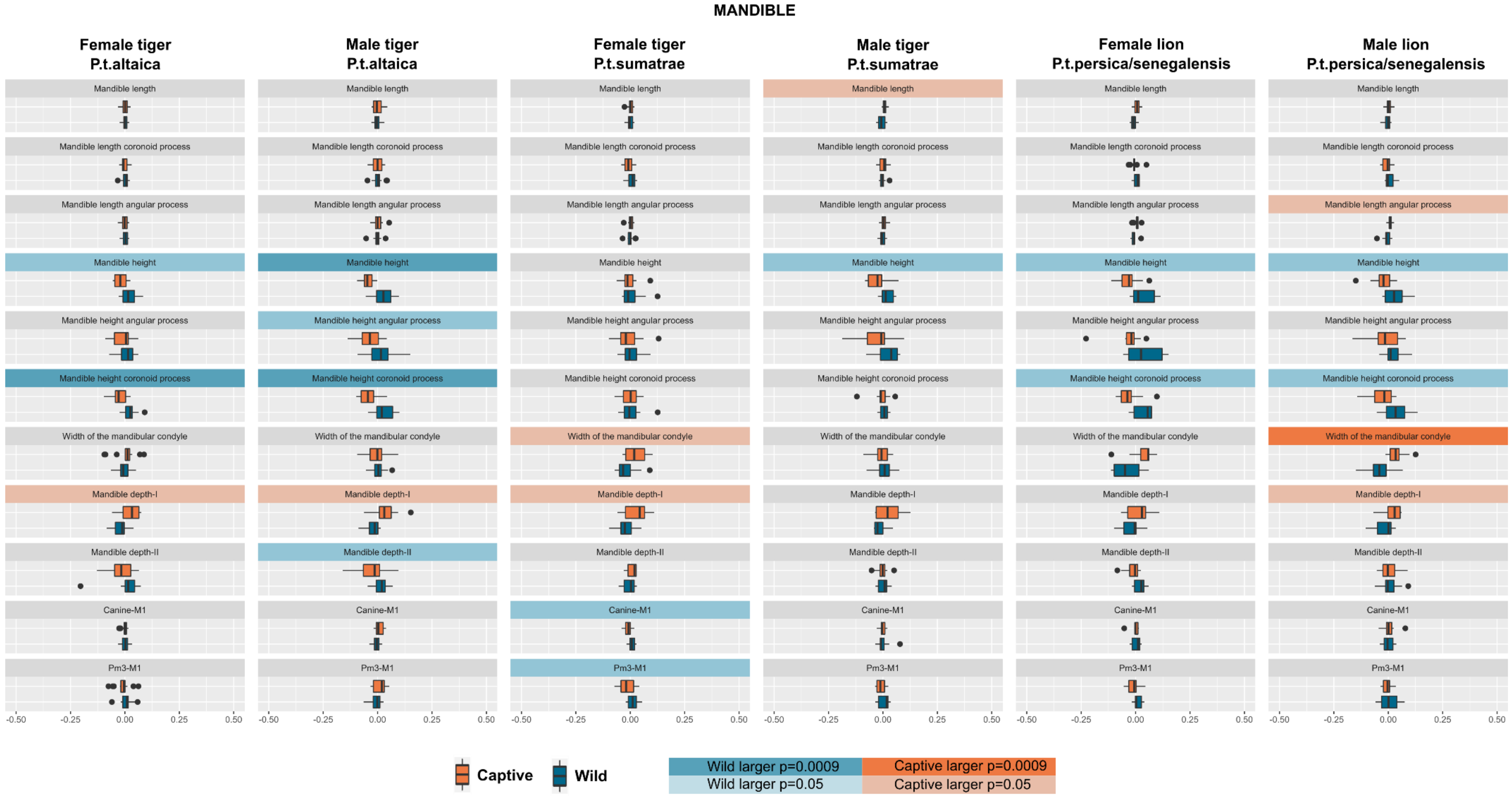

Figure S12: Variation of size independent (scaled) variables by captivity status for measurements of the mandible when the data is split by nominal grouping. Measurements larger than zero are larger than average, and measurements smaller than zero are below average for a given sized skull. Measurements which differ significantly by captivity status are highlighted by which population mean is larger. Significance is determined using t-tests (Table S2) based on values of 0.05 and after a Bonferroni Correction, 0.0009.

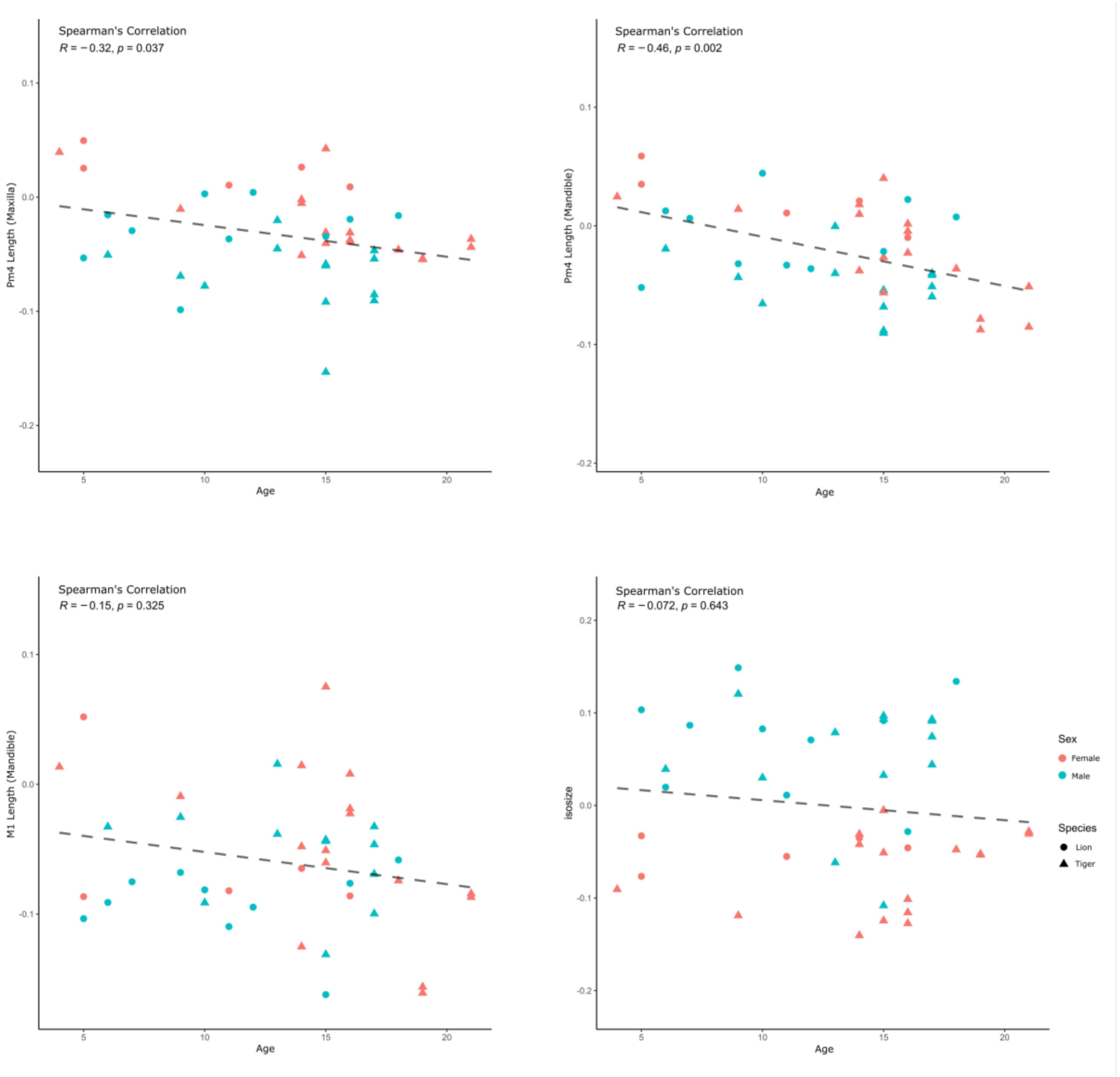

Figure S13: Measurements of tooth length and isosize against captive lions and tigers of known age. There is no relationship between isosize and age (Spearman's correlation p=0.643). Tooth length decreases significantly with age in Pm4 length (maxilla) p=0.037 and in Pm4 length (mandible) p=0.002. Whilst not significant, M1 length (mandible) also decreases with age (p=0.325). This suggests that tooth wear may have affected tooth measurements, and that smaller tooth size in captive big cats may in part be explained by their longer life expectancy when compared with those from the wild.

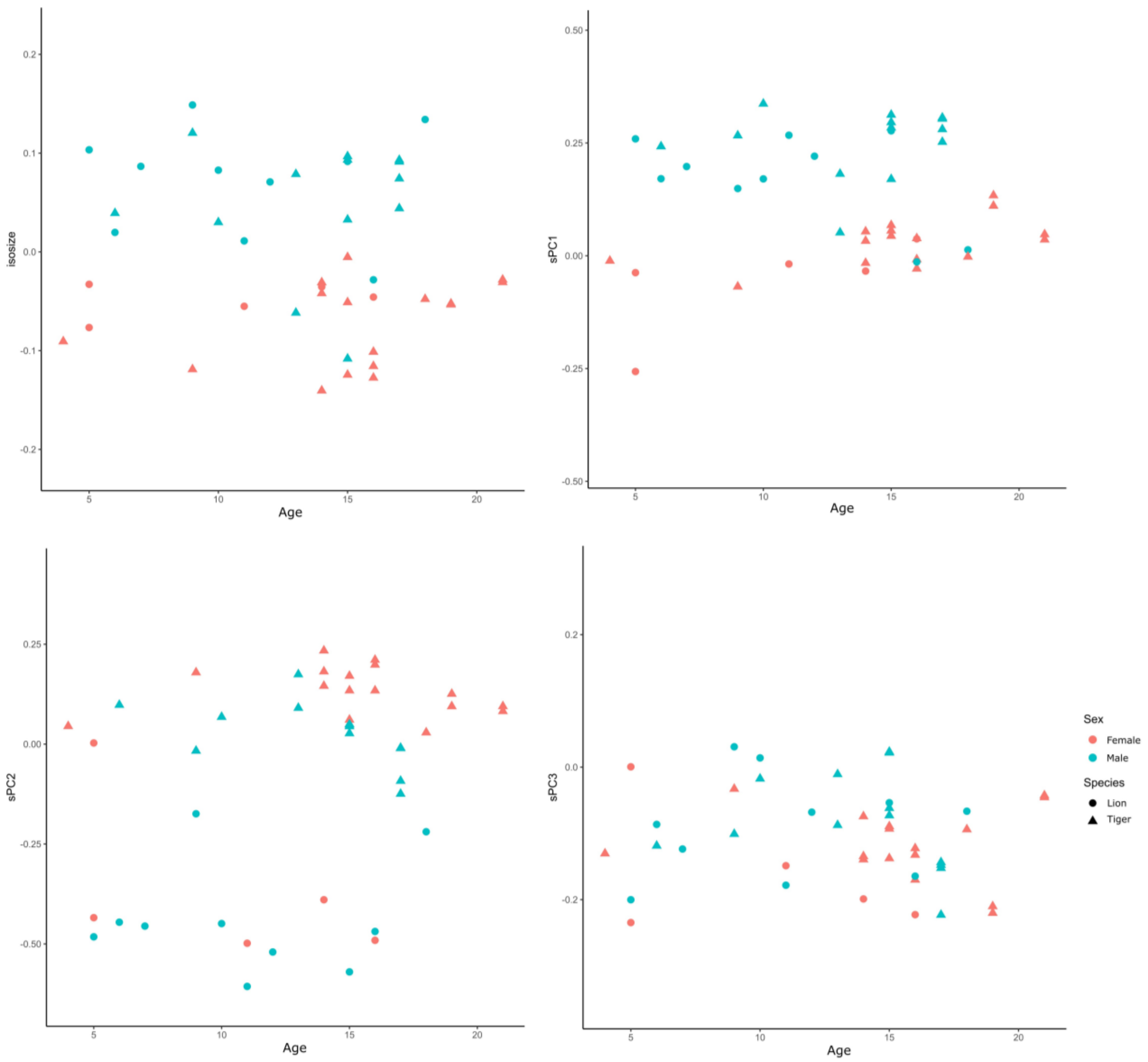

Figure S13: Shape principal components and isosize against captive lions and tigers of known age. There is no apparent relationship between size or shape and age in the captive adult specimens analysed. sPC3 was discriminatory between captive and wild populations of big cats (*Figure 2*), yet shows no relationship with age. It is therefore unlikely that any age disparity between captive and wild populations has influenced our findings.
